# ICU survival and need of renal replacement therapy with respect to AKI duration in critically ill patients

**DOI:** 10.1186/s13613-018-0467-6

**Published:** 2018-12-17

**Authors:** A. S. Truche, S. Perinel Ragey, B. Souweine, S. Bailly, L. Zafrani, L. Bouadma, C. Clec’h, M. Garrouste-Orgeas, G. Lacave, C. Schwebel, F. Guebre-Egziabher, C. Adrie, A. S. Dumenil, Ph. Zaoui, L. Argaud, S. Jamali, D. Goldran Toledano, G. Marcotte, J. F. Timsit, M. Darmon

**Affiliations:** 1 UMR 1137 - IAME Team 5 – DeSCID : Decision SCiences in Infectious Diseases, Control and Care, Inserm/Paris Diderot University, Sorbonne Paris Cité, Paris, France; 2 Medical Intensive Care Unit, Grenoble University Hospital, Grenoble 1 University, U823, La Tronche, France; 3 Nephrology Dialysis Renal Transplantation, Grenoble University Hospital, La Tronche, France; 4 Medical Intensive Care Unit, Croix Rousse Hospital, Lyon University Hospital, Lyon, France; 5 Medical Intensive Care Unit, Gabriel Montpied University Hospital, Clermont-Ferrand, France; 6 Medical Intensive Care Unit, AP-HP, Saint Louis Hospital, Paris, France; 7 Medicine University, Paris 7 University, Paris, France; 8 Medical and Infectious Diseases Intensive Care Unit, AP-HP, Bichat Hospital, Paris Diderot University, 75018 Paris, France; 9 Intensive Care Unit, AP-HP, Avicenne Hospital, Paris, France; 10 Medicine University, Paris 13 University, Bobigny, France; 11 Intensive Care Unit, Saint Joseph Hospital Network, Paris, France; 12 Medicine University, Paris Descartes University, Sorbonne Cite, Paris, France; 13 Medical Intensive Care Unit, André Mignot Hospital, Versailles, France; 14 Physiology Department, Cochin University Hospital, Assistance Publique, Hôpitaux de Paris (AP-HP), Paris Descartes University des, Sorbonne Cite, Paris, France; 15 Medical-Surgical Intensive Care Unit, AP-HP, Antoine Béclère University Hospital, Clamart, France; 16 Medical Intensive Care Unit, Edouard Herriot University Hospital, Lyon, France; 17 Critical Care Medicine Unit, Dourdan Hospital, Dourdan, France; 18 Intensive Care Unit, Gonesse Hospital, Gonesse, France; 19 Surgical ICU, Edouard Herriot University Hospital, Lyon, France; 20 ECSTRA Team, Biostatistics and Clinical Epidemiology, UMR 1153 (Center of Epidemiology and Biostatistics Sorbonne Paris Cité, CRESS), INSERM, Paris, France

**Keywords:** Acute kidney injury, Intensive care unit, Renal recovery, Renal replacement therapy, Epidemiology and outcome

## Abstract

**Background:**

Transient and persistent acute kidney injury (AKI) could share similar physiopathological mechanisms. The objective of our study was to assess prognostic impact of AKI duration on ICU mortality.

**Design:**

Retrospective analysis of a prospective database via cause-specific model, with 28-day ICU mortality as primary end point, considering discharge alive as a competing event and taking into account time-dependent nature of renal recovery. Renal recovery was defined as a decrease of at least one KDIGO class compared to the previous day.

**Setting:**

23 French ICUs.

**Patients:**

Patients of a French multicentric observational cohort were included if they suffered from AKI at ICU admission between 1996 and 2015.

**Intervention:**

None.

**Results:**

A total of 5242 patients were included. Initial severity according to KDIGO creatinine definition was AKI stage 1 for 2458 patients (46.89%), AKI stage 2 for 1181 (22.53%) and AKI stage 3 for 1603 (30.58%). Crude 28-day ICU mortality according to AKI severity was 22.74% (*n* = 559), 27.69% (*n* = 327) and 26.26% (*n* = 421), respectively. Renal recovery was experienced by 3085 patients (58.85%), and its rate was significantly different between AKI severity stages (*P* < 0.01). Twenty-eight-day ICU mortality was independently lower in patients experiencing renal recovery [CSHR 0.54 (95% CI 0.46–0.63), *P* < 0.01]. Lastly, RRT requirement was strongly associated with persistent AKI whichever threshold was chosen between day 2 and 7 to delineate transient from persistent AKI.

**Conclusions:**

Short-term renal recovery, according to several definitions, was independently associated with higher mortality and RRT requirement. Moreover, distinction between transient and persistent AKI is consequently a clinically relevant surrogate outcome variable for diagnostic testing in critically ill patients.

**Electronic supplementary material:**

The online version of this article (10.1186/s13613-018-0467-6) contains supplementary material, which is available to authorized users.

## Introduction

One out of two intensive care unit (ICU) patients will experience an acute kidney injury (AKI) during his ICU stay [1]. This complication is responsible for a high burden: drastic short- and long-term mortality increase [2, 3] and persistent renal dysfunction [4]. Classifications such as the risk, injury, failure, loss of kidney function, and end-stage renal disease (RIFLE) one [5], shortly followed by acute kidney injury network (AKIN) [6] and at last by kidney disease: improving global outcomes (KDIGO) were developed in order to allow a better description of AKI spectrum [7]. They provided a consensual definition for AKI diagnosis and staging and enabled comparability between studies. However, these classifications do not integrate AKI duration in their criteria. The ADQI proposed a classification according to timing of recovery, relying, however, on expert opinion and requiring validation [8].

Transient AKI was classically thought to be due to pre-renal azotemia, whereas persistent AKI was considered as a consequence of acute tubular necrosis (ATN) [9]. These last years, several studies have contributed to question this paradigm [10–13]. AKI duration appears rather to be linked to AKI severity than to distinct physiopathological mechanism [11]. In a previous study [14] with unselected ICU patients, persistent AKI was far more frequent than transient AKI, associated with more severe AKI and more likely to fulfill both serum creatinine and diuresis criteria. When AKI severity was introduced into the model, the association between AKI duration and patients’ outcome disappeared, leading to the hypothesis that transient and persistent AKI could share similar pathophysiological mechanisms. However, our results could have failed to demonstrate an association between AKI duration and outcome due to an insufficient statistical power. Additionally, in this previous study, time-dependent nature of renal recovery (i.e., dead patients will never recover from their AKI) was only partly taken into account. Thus, a new and larger study was performed in a prospective multicentric French ICU cohort. By using a cause-specific model, the aim was to take into account competitive risk arising from discharged alive patients and time-dependent nature of renal recovery.

The primary objective of this study was to assess prognostic impact of AKI duration on 28-day ICU mortality. Secondary objective was to assess relationship between renal recovery at specific time frames and need for renal replacement therapy.

## Patients and methods

### Study population

Patients of the OUTCOMEREA™ cohort were included in the study if they suffered from AKI at ICU admission during the period ranging from 1996 to 2015. OUTCOMEREA™ database has already been described in some details [15] (see Additional file 1: quality of the database). Briefly, patients over 16 years of age admitted to 23 French ICUs were included in this retrospective analysis of an observational prospective multicenter cohort. Patients’ demographic, clinical and biological data were collected at baseline and daily during their ICU stay. The database was approved by CCTIRS and CNIL (number 999262), respectively the French Advisory Committee for Data Processing in Health Research and the French Informatics and Liberty commission. The study was approved by the ethics committee of Clermont-Ferrand (number 5891), France, and was performed in accordance with the Declaration of Helsinki.

Exclusion criteria were: chronic kidney disease at ICU admission and absence of creatinine value recorded in the database on the first day of ICU stay. In case of readmission, only the first ICU stay was considered.

### Definitions

AKI at ICU admission was defined according to KDIGO classification [7]. Since 6- and 12-h diureses were not available in the database, only the creatinine component of this classification was used. Similarly, initial AKI and changes in renal dysfunction severity were assessed according to KDIGO creatinine criteria.

Baseline creatinine value was estimated via inverse Modification of Diet in Renal Disease (MDRD) formula, considering normal baseline GFR (75 ml/min/1.73 m^2^) [7] for all included patients.

Renal recovery was considered as a decrease of at least one KDIGO class compared to the previous day. For sensitive analysis purpose, an alternative definition considering renal recovery as full recovery of AKI according to KDIGO criteria was used.

Patients requiring RRT were classified as AKI stage 3 and considered as being weaned from RRT only if not requiring RRT for at least 5 days.

Discharge alive was defined as survival at discharge from the ICU.

Initial severity was assessed according to Simplified Acute Physiology Score II (SAPS II) [16] and Sequential Organ Failure Assessment (SOFA) Score [17]. Septic shock was defined according to the Third International Consensus Definitions for Sepsis and Septic Shock (Sepsis-3) [18].

Chronic kidney disease was defined according to usual definitions [19] and reported in the dataset by investigator using ICD-10 code (N18-N18, N18.0, N18.8, N18.9, I12.0, N11-N11, N11.0, N11.1, N11.8, N11.9, N03-NO3, N03.0, N03.1, N03.2, N03.3, N03.4, N03.5, N03.6, N03.7, N03.8, N03.9, N19, N08.3).

### Statistical analysis

Quantitative variables are presented as median and interquartile range and compared between groups with the Wilcoxon test. Qualitative variables are presented as frequency and corresponding percentage and compared with the Chi-square test.

In our first model, we aimed to assess the impact of renal recovery on 28-day ICU mortality. In this situation, discharge alive was considered as a competing event for the outcome. Cause-specific models are survival models used in the presence of competing risk. They allow fitting separate Cox model for each endpoint. Hence, cause-specific hazard ratio (= CSHR) obtained for the two endpoints can be concurrently interpreted for each model [20]. Renal recovery status was introduced as a time-dependent variable [21]. Variables identified in the literature as potential confounding factors were introduced into the model for adjustment. Baseline variables were: shock and initial AKI severity class. Time-dependent variables were Sequential Organ Failure Assessment (SOFA) score components, except renal one, and nephrotoxic drug administration in the five previous days. Subgroup analyses were conducted in patients suffering from diabetes, hypertension or septic shock during the first 24 h.

A sensitivity analysis was conducted considering a threshold of 3 days to distinguish transient and persistent AKI: a transient AKI was a renal recovery occurring within the first 3 days; otherwise, it was a persistent AKI. Only patients still alive and in ICU for at least 72 h were kept in the analysis, since the transient or persistent nature of AKI could not be determined before. A Cox model was used to assess the impact of AKI duration on 28-day ICU mortality, with adjustment on the worst value of the confounding risk factors during the first 3 days of ICU stay for patients with persistent AKI and before renal recovery for patients with transient AKI.

Last, performances of various definitions of persistent AKI (defined as a lack of renal recovery between day 2 and day 7) in predicting need for renal replacement therapy during ICU stay were evaluated in patients staying at least 8 days in ICU.

A *P* value of 0.05 was retained for statistical significance.

All statistical analyses were conducted with SAS 9.4 (SAS Institute Inc., Cary, NC, USA).

## Results

### Initial characteristics

**Fig. 1:**
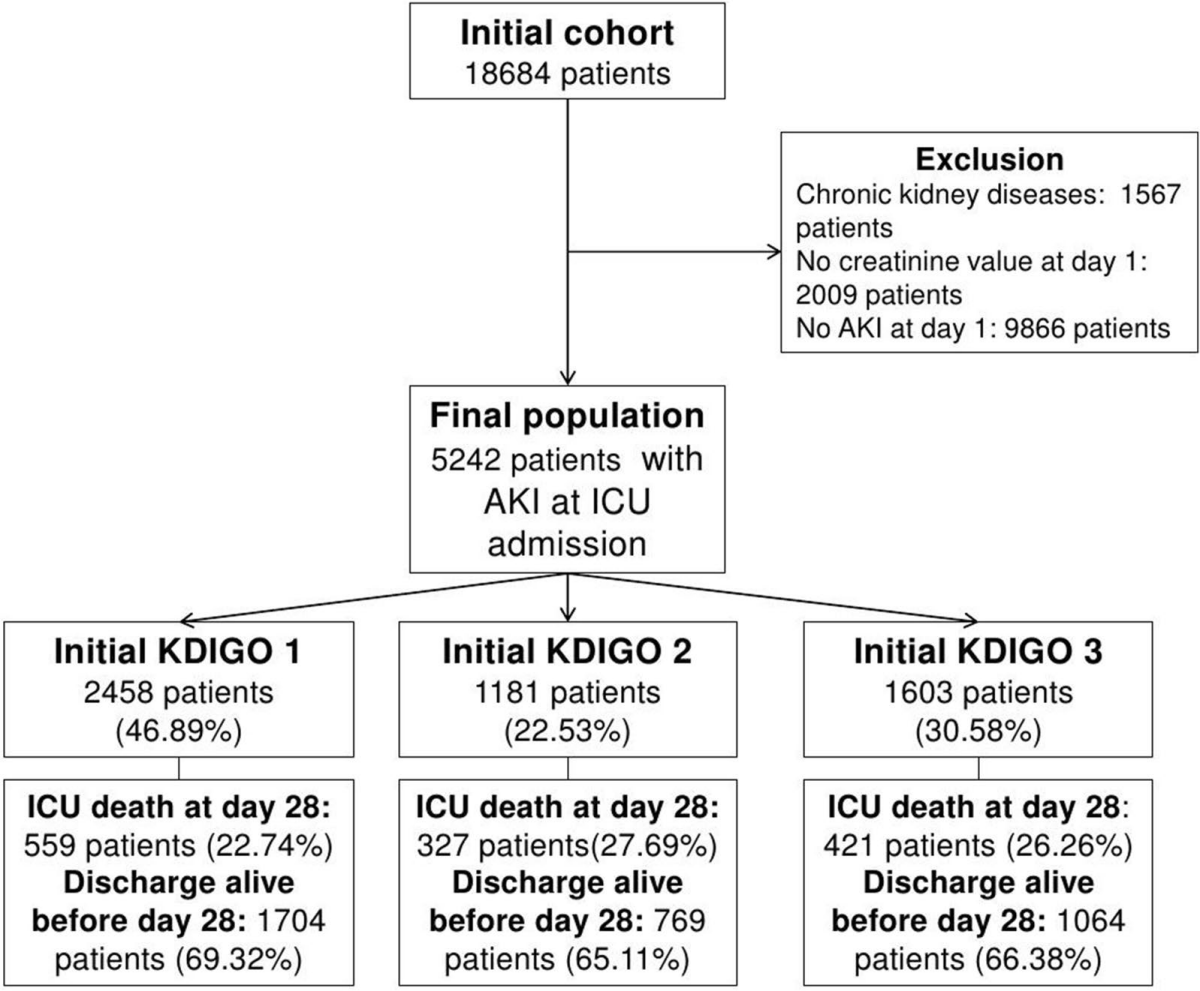
Flowchart. *AKI* acute kidney injury; *KDIGO* kidney disease: improving global outcomes; *ICU* intensive care unit

Of the 18,684 patients screened, 5242 patients were finally included in the study (Fig. 1).

The initial severity according to KDIGO definition was AKI stage 1 for 2458 patients (46.89%), AKI stage 2 for 1181 (22.53%) and AKI stage 3 for 1603 (30.58%) (Table 1).

**Table 1:** Patients initial characteristics in the overall population and according to kidney disease: improving global outcomes definition at day 1

Variable	All (*n* = 5242)	AKI stage 1 (*n* = 2458)	AKI stage 2 (*n* = 1181)	AKI stage 3 (*n* = 1603)	*P* value*
Patients characteristics					
Sex (male)	3176 (60.59)	1591 (64.73)	663 (56.14)	922 (57.52)	< .01
Age (years)	70.2 [58.0–78.9]	70.5 [58.4–78.9]	72.5 [60.9–80.1]	68.0 [56.2–77.4]	< .01
SAPS II	50 [37–67]	46 [35–61]	52 [39–69]	54 [41–72]	< .01
SOFA^a^	5 [3, 10]	5 [3–8]	6 [3–8]	5 [3–9]	< .01
Pulmonary insufficiency immuno	651 (12.42)	373 (15.17)	147 (12.45)	131 (8.17)	< .01
Suppression	946 (18.05)	389 (15.83)	223 (18.88)	334 (20.84)	< .01
AKI risk factors					
Cardiac insufficiency	961 (18.33)	434 (17.66)	247 (20.91)	280 (17.47)	0.03
Cirrhosis	344 (6.56)	147 (5.98)	79 (6.69)	118 (7.36)	0.22
Diabetes	1098 (20.95)	506 (20.59)	252 (21.34)	340 (21.21)	0.83
Nephrotoxic^b^	1355 (25.85)	611 (24.86)	322 (27.27)	422 (26.33)	0.26
Contrast agent	330 (6.30)	167 (6.79)	67 (5.67)	96 (5.99)	0.36
Aminoside	954 (18.20)	421 (17.13)	235 (19.90)	298 (18.59)	0.11
Glycopeptide	404 (7.71)	170 (6.92)	94 (7.96)	140 (8.73)	0.10
Reason for ICU admission					
Admission					< .01
Medical	4031 (77.15)	1870 (76.23)	919 (78.21)	1242 (77.77)	
Emergency surgery	827 (15.83)	356 (14.51)	197 (16.77)	274 (17.16)	
Scheduled surgery	367 (7.02)	227 (9.25)	59 (5.02)	81 (5.07)	
Shock	2148 (40.98)	862 (35.07)	554 (46.91)	732 (45.66)	< .01
ARF	1183 (22.57)	706 (28.72)	272 (23.03)	205 (12.79)	< .01
Hospital stay before ICU admission (days)	0 [0;3]	0 [0;2]	0 [0;3]	0 [0;3]	0.81
Outcome					
AKI duration	2 [1–5]	2 [1–4]	2 [1–4]	3 [2–6]	< .01
Day-28 renal recovery	3085 (58.85)	1613 (65.62)	741 (62.74)	731 (45.60)	< .01
Day-28 mortality	1307 (24.93)	559 (22.74)	327 (27.69)	421 (26.26)	< .01
Discharge alive at Day 28	3537 (67.47)	1704 (69.32)	769 (65.11)	1064 (66.38)	0.03
RRT requirement during ICU stay	1336 (25.49)	275 (11.19)	179 (15.16)	882 (55.02)	< .01

Qualitative variables are presented as frequency (and corresponding percentage), quantitative variables as median (interquartile range)

*KDIGO* kidney disease: improving global outcomes; *SAPS* Simplified Acute Physiology Score; *SOFA* Sequential Organ Failure Assessment; AKI, Acute kidney injury; ICU, intensive care unit; ARF, Acute respiratory failure; RRT, Renal replacement therapy

* Comparison between the three tested groups

^a^Except renal component

^b^The day of admission

Median age in the population was 70.2 years [58.0–78.9]. Diabetes was the most frequent comorbidity (20.95%). Population initial severity was 50 [37–67] assessed by SAPSII score and 5 [3–10] by SOFA score. The majority of the included patients were admitted due to a medical condition (77.15%). Shock and respiratory failure were the main organ dysfunctions at ICU admission (respectively, 40.98% and 22.57% of the included patients). Overall, 1355 patients (25.85%) had received nephrotoxic agent at ICU admission and aminoglycoside was the main nephrotoxic in this study (18.20%). A total of 330 (6.30%) patients received iodinated contrast agents.

### Renal recovery impact on 28-day ICU mortality

Crude 28-day ICU mortality according to AKI severity was 22.74% (*n* = 559), 27.69% (*n* = 327) and 26.26% (*n* = 421) for patients with AKI stages 1, 2 and 3, respectively.

Rate of renal recovery was significantly different between AKI severity stages (65.62, 62.74 and 45.60% for AKI stages 1, 2 and 3, respectively; *P* < 0.01). AKI lasted longer in patients with AKI stage 3 (3 days [2–6] vs. 2 days [1–4] for stage 1 and 2). Among patients who experienced renal recovery, 431 (13.97%) died, versus 876 (40.61%) patients without recovery. Maximum AKI stage was stage 1 for 1829 patients (34.89%), stage 2 for 1155 (22.03%) and stage 3 for 2258 (43.08%) patients (*P* < 0.01). After adjustment for confounding factors, 28-day ICU mortality was independently lower in patients experiencing renal recovery [CSHR 0.54 (95% CI 0.46–0.63), *P* < 0.01; Table 2], whereas 28-day ICU discharge was significantly higher [CSHR 1.85 (95% CI 1.72–1.99), *P* < 0.01; Table 2].

**Table 2:** Discharge alive and 28-day mortality cause-specific model according to renal recovery defined as a decrease of at least one kidney disease: improving global outcomes class compared to the previous day

Parameter	Discharge alive		28-Day mortality	
	CSHR (95% CI)	*P* value	CSHR (95% CI)	*P* value
Shock at admission	0.90 (0.84–0.97)	0.01	1.27 (1.12–1.43)	< .01
KDIGO Day 1		0.68		0.25
1	ref		ref	
2–3	1.01 (0.95–1.08)		1.07 (0.95–1.20)	
Nephrotoxic in the 5 previous days^a^	0.79 (0.73–0.85)	< .01	0.93 (0.82–1.05)	0.21
Daily Cardiological SOFA^a^		< .01		< .01
0	ref		ref	
1	0.88 (0.81–0.95)	< .01	2.07 (1.68–2.56)	< .01
2	0.78 (0.66–0.92)	< .01	2.19 (1.58–3.02)	< .01
3	0.54 (0.49–0.60)	< .01	1.86 (1.50–2.30)	< .01
4	0.49 (0.44–0.54)	< .01	3.09 (2.53–3.76)	< .01
Daily Liver SOFA^a^		< .01		0.03
0	ref		ref	
1	0.94 (0.84–1.05)	0.24	1.01 (0.83–1.22)	0.93
2	0.77 (0.68–0.88)	< .01	1.08 (0.90–1.29)	0.42
3	0.57 (0.44–0.75)	< .01	1.23 (0.93–1.63)	0.14
4	0.70 (0.55–0.89)	< .01	1.55 (1.18–2.04)	< .01
Daily Respiratory SOFA^a^		< .01		< .01
0	ref		ref	
1	0.73 (0.67–0.81)	< .01	0.79 (0.65–0.97)	0.02
2	0.67 (0.62–0.73)	< .01	0.84 (0.70–1.00)	0.05
3	0.41 (0.36–0.46)	< .01	1.15 (0.96–1.37)	0.12
4	0.36 (0.28–0.45)	< .01	1.78 (1.43–2.22)	< .01
Daily Coagulation SOFA^a^		0.13		< .01
0	ref		ref	
1	0.92 (0.84–1.01)	0.07	0.96 (0.80–1.13)	0.60
2	0.95 (0.86–1.05)	0.30	1.05 (0.88–1.26)	0.57
3	0.91 (0.79–1.05)	0.21	1.39 (1.13–1.70)	< .01
4	0.79 (0.60–1.03)	0.08	1.95 (1.52–2.52)	< .01
Daily Neurological SOFA^a^		< .01		< .01
0	ref		ref	
1	0.68 (0.61–0.77)	< .01	1.39 (1.13–1.69)	< .01
2	0.61 (0.54–0.69)	< .01	1.56 (1.28–1.89)	< .01
3	0.48 (0.42–0.55)	< .01	1.99 (1.64–2.40)	< .01
4	0.36 (0.30–0.43)	< .01	3.99 (3.39–4.70)	< .01
Renal recovery^a^	1.85 (1.72–1.99)	< .01	0.54 (0.46–0.63)	< .01

*CSHR* cause-specific hazard ratio; *CI* confidence interval; *KDIGO* kidney disease: improving global outcomes; *SOFA* Sequential Organ Failure Assessment

^a^All time-dependent variables values are those of the previous day

These results were consistent among the different subgroups considered (Additional file 1: Table E1). In particular, among patients with septic shock, renal recovery occurrence was associated with a dramatically increase in discharge alive status (CSHR: 2.71 (95% CI 2.32–3.16), *P* < 0.01).

### Sensitivity analysis

A sensitivity analysis was performed assessing influence of renal recovery when defined by full recovery of AKI. According to this definition, 2184 (41.66%) patients experienced renal recovery. Rate of renal recovery was significantly different across class of renal dysfunction severity [1432 (58.26%), 418 (35.39%) and 334 (20.84%), respectively, in patients with AKI stages 1, 2 and 3; *P* < 0.01)]. Using this definition, renal recovery remains independently associated with decreased 28-day ICU mortality [CSHR 0.55 (95% CI 0.47–0.66), *P* < 0.01; Additional file 1: Table E2].

**Fig. 2:**
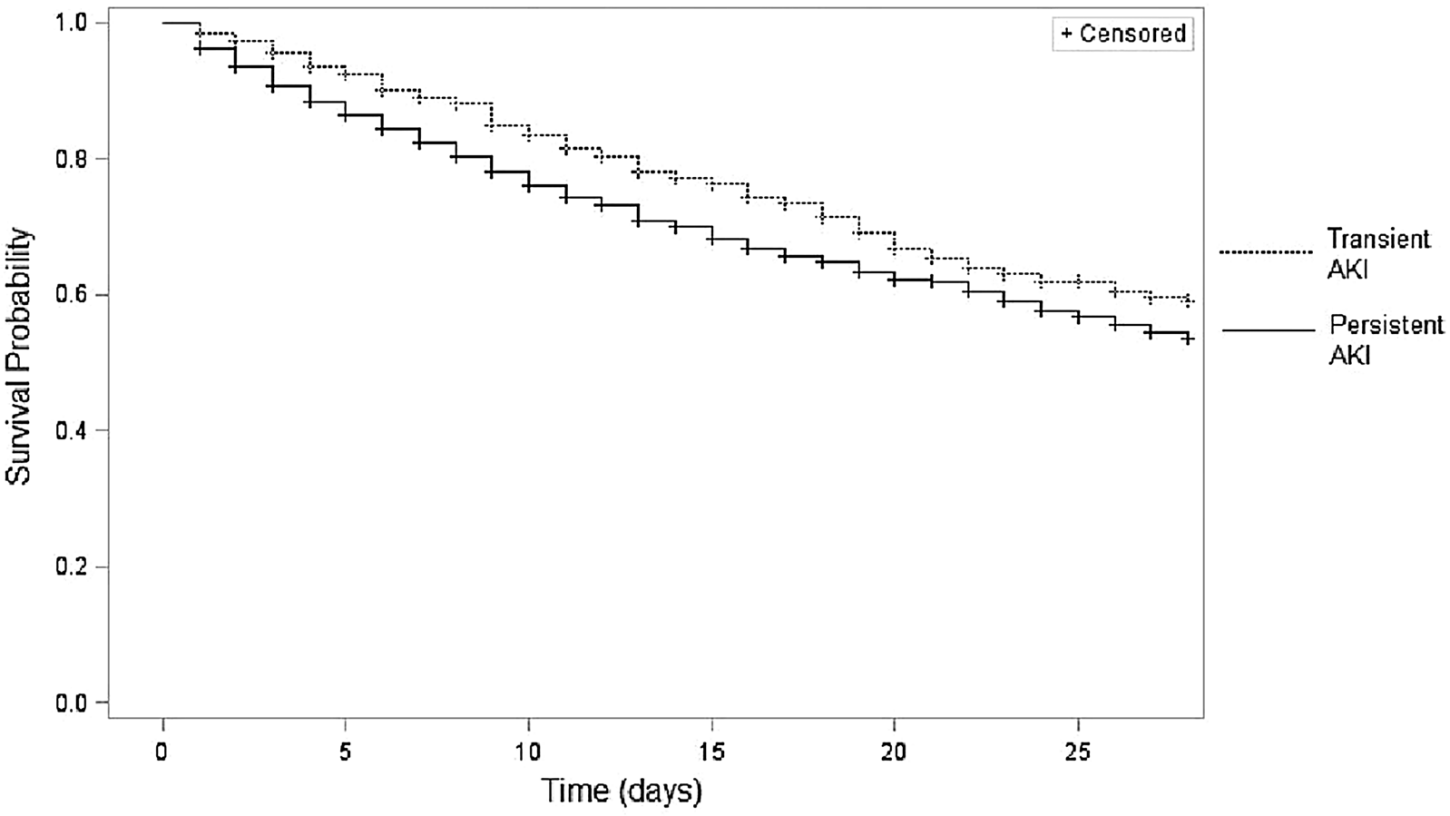
Survival curve according to persistence of acute kidney injury defined as an absence of renal recovery occurring within the first 3 days. *n* = 3584, Log rank < 0.01. *AKI* acute kidney injury

Last, in order to compare our results with previous studies in this field, influence of recovery within 72 h was assessed. After adjustment for confounding factors and AKI maximum severity in the first 3 days, transient AKI was independently associated with a decreased 28-day ICU mortality [HR 0.80 (95% CI 0.67–0.95), *P* = 0.01; Additional file 1: Table E3]. Corresponding survival curve is reported in Fig. 2.

### AKI duration and prediction of RRT requirement

**Fig. 3:**
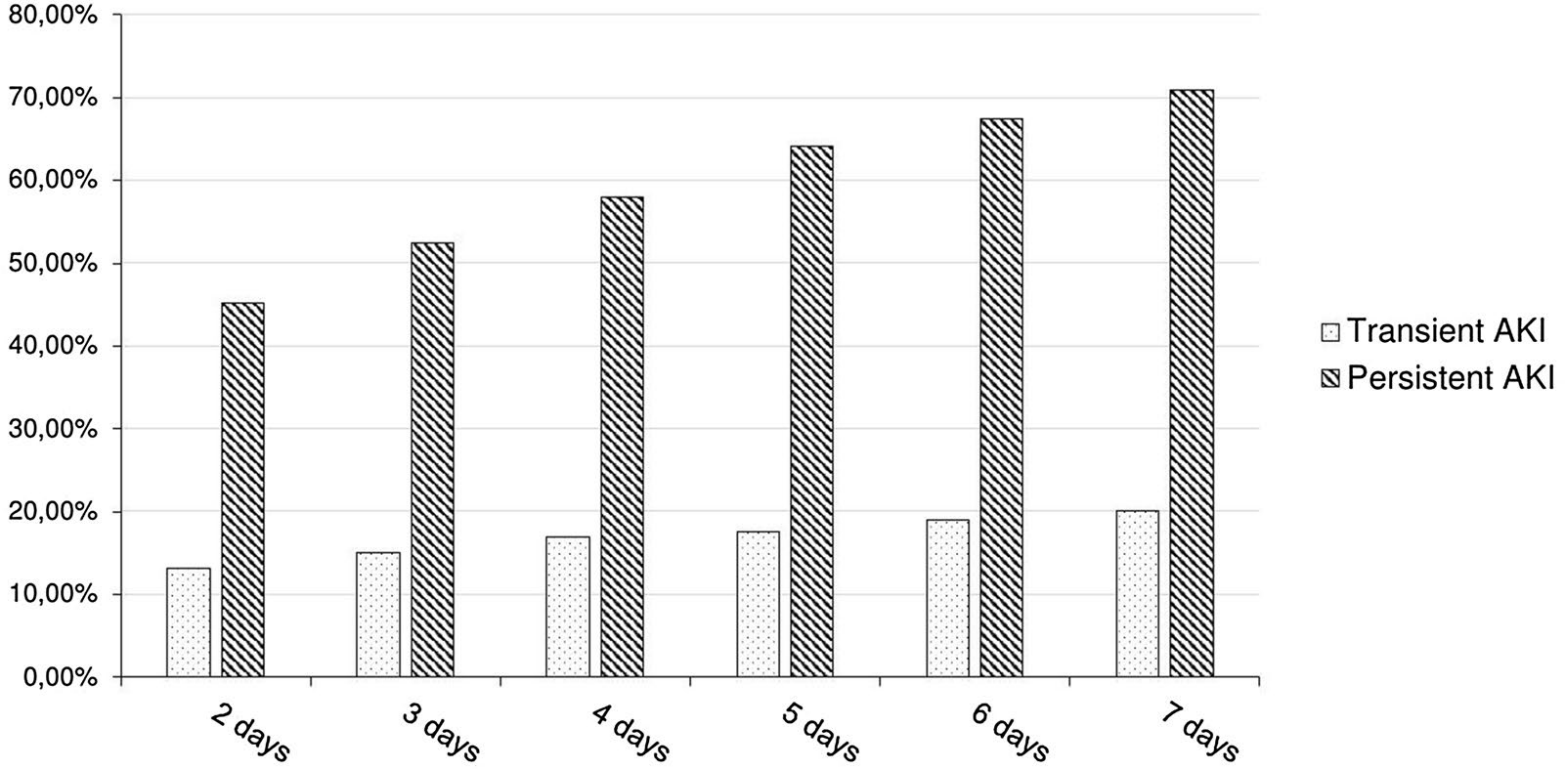
Renal replacement therapy requirement according to persistent and transient acute kidney injury defined at various time frames: *n* = 2055. *AKI* acute kidney injury

Rate of renal replacement therapy according to renal recovery at various time frames is reported in Fig. 3. Since day-2 threshold, persistent AKI appeared as a strong predictor of RRT requirement. Sensitivity decreased when choosing a higher threshold (from 93% at day 2 to 67% at day 7), whereas specificity increased (from 30% at day 2 to 83% at day 7).

## Discussion

To the best of our knowledge, this is the first study based on a large multicentric ICU cohort assessing the prognostic impact of renal recovery when taking into account its time-dependent nature. Reversibility of AKI appeared as a strong predictor of enhanced survival in a time-dependent cause-specific model while considering discharge alive as a competing event. Secondly, an incremental AKI duration was associated with a poorer prognosis when persistent AKI was defined as a lack of renal recovery within 3 days, as compared to transient AKI. Lastly, RRT requirement was drastically increased in patients experiencing persistent AKI whichever threshold between 2 and 7 days was chosen to delineate transient and persistent AKI.

In our study, lack of renal recovery was associated with a significantly higher mortality and this result was confirmed in the different subgroups of patients at higher risk of kidney injury. Interestingly, this effect persisted even after adjustment on initial AKI severity. Up until now, studies based on recent AKI classifications and aiming to assess AKI prognostic impact on short- and long-term mortality have mainly focused on the effect of the maximum severity class reached [1, 22, 23] or AKI severity at ICU admission [3]. Whichever classification was considered, AKI occurrence was associated with a decreased survival. Of note, several studies found a similar risk for patients experiencing AKI-injury and AKI-failure class according to RIFLE classification [3, 24, 25], indicating that the maximum mortality risk was potentially reached as soon as a patient experiences AKI-injury class.

Several studies suggested that AKI duration and severity could be associated [11, 26]. One of the striking results of our previous study was that patients with persistent AKI, defined as an absence of renal recovery within 3 days, were more likely to meet both oliguria and serum creatinine elevation criteria for AKI and tended to experience more severe AKI than patients with transient AKI [14]. In a large cohort of 30,000 patients, short- and long-term outcomes appeared to be dramatically worse when a severity stage was reached by both criteria [27]. These findings were, however, unadjusted for time dependency of renal recovery [27].

Transient and persistent AKI were classically thought to be due to distinct physiopathological mechanisms, namely pre-renal azotemia and acute tubular necrosis [9]. This concept has been challenged these last years by experimental and clinical findings demonstrating the paucity of ATN features on renal biopsy [10] or its focal nature [12]. Urinary biomarker seemed also inefficient to predict an early renal recovery [13, 26, 28]. Hence, in accordance with these recent findings, transient and persistent AKI should rather be considered as a continuum of a same pathology with increasing severity [29].

Surprisingly, even though some data suggested that AKI duration could be a marker of severity, its impact, independent of those of AKI severity, and consequences of AKI reversible nature have poorly been studied in the literature. In a large cohort of 20,126 patients, Uchino et al. [30] showed an increasing mortality with AKI duration, this risk existing even for 1-day-lasting AKI. Similar results were found in postoperative contexts [31] and in ICU settings [26]. Interestingly, in a large multicentric cohort of diabetic patients who underwent non-cardiac surgery, in each strata of AKI duration, mortality was no longer influenced by AKIN severity classes [32]. These studies are yet insufficient to conclude due to consequent limitations concerning study population and limiting external generalizability of their conclusions (specific ICU patients’ subset [33, 34], use of monocentric cohorts [33, 35]). In a previous study [14], by including unselected critically ill patients from a multicentric cohort, we were able to demonstrate a lower hospital survival in the presence of persistent AKI, but this effect disappeared after adjustment on AKI severity. However, these results could have been influenced by a lack of statistical power and lead us to conduct another trial based on the high-quality multicentric cohort OUTCOMEREA™.

Statistical tools used in previous studies are also questionable. A consequent methodological limitation is linked to competing risk resulting from patients discharged alive from ICUs. In ICU settings, discharge alive is an informative censoring because censored patients are different from patients staying in ICU. It modifies the probability to observe the outcome, i.e., ICU death in the population staying in the unit. Standard survival methods in this case can no longer be used [36]. By using a cause-specific model, we were able to bypass this limitation and to estimate simultaneously a cause-specific hazard ratio for each outcome, ICU mortality and discharge alive. Another limitation arises from assumption in most studies that AKI reversibility was known since admission, even in largest trials [27], leading to a time-dependent bias [37]. In a study of Kellum et al. [38], patients without renal recovery had a decreased survival when compared to patients with partial or full recovery. In a second study, they identified several recovery patterns according to the delay before renal recovery and the occurrence of a relapse with or without a subsequent recovery, which were associated with different 1-year prognoses [39]. These findings were, however, probably influenced by the time dependency of renal recovery; patients dying before the occurrence of renal recovery will never experience this event. As a consequence, the absence of renal recovery can falsely be associated with mortality. Thus, in our study, this variable was introduced as time dependent. Another advantage to use cause-specific model is to adjust on confounding factors, i.e., patients’ severity represented by SOFA score component and nephrotoxic exposure, not only not only considering them at baseline but taking into account their evolution with respect to time sequence. Lastly, as in our previous work [14], persistent AKI was associated with a much higher rate of RRT requirement than patients experiencing a renal recovery. A threshold between day 2 and day 7 for defining AKI duration could thus be used as a surrogate marker for further need of RRT during ICU stay. The threshold should be chosen according to clinician preference, a greater sensitivity (day 2) or specificity (day 7).

Recently, studies have pointed out the importance of the renal recovery definition considered [40–42]. In a recent position paper, the ADQI group recommends defining recovery as full renal recovery, early recovery being defined by recovery within 48 h and acute kidney disease by a failure to recover within 7 days [8]. Although our findings confirm full recovery to be associated with outcome, they also demonstrate that incomplete recovery, as defined by decrease of at least one KDIGO class, is also associated with improved outcome. This finding may help to refine definition proposed by ADQI group and is a plea to further research to validate this definition. But more importantly, this finding is in keeping with known delay between improved glomerular filtration rate and serum creatinine decrease [43] and suggests that reduction of at least one KDIGO severity class may be a clinically relevant objective.

Several limitations in our study should be acknowledged. First of all, baseline creatinine was not available, so we had to estimate this value thanks to MDRD equation. Even if this method is suggested in KDIGO [7], it can lead to an excess in AKI diagnosis and reduce renal recovery probability [44]. Secondly, AKI staging was only based on creatinine criteria because hourly diuresis was not available in our database. As previously explained, reaching an AKI stage with both criteria could be associated with more severe AKI [14, 27] and it could have been interesting to include this data into the model. Due to muscle loss during ICU stay, renal recovery could have been over diagnosed. Only the first AKI episode was taken into account, and no conclusion could be inferred concerning the influence of a further relapse. The endpoint for analysis was limited at day 28. Hence, at this time, 92% of the tested patients either died or were discharged precluding further follow-up. Whether definition of renal recovery may influence longer-term outcome is unanswered by this study and may deserve to be further studied. Lastly, although multicentric, our population was mainly admitted for medical condition, limiting potentially the extension of these conclusions to surgical patients.

## Conclusions

This study, taking time dependency of renal recovery into account confirms the prognostic impact of early renal recovery and the clinical relevancy of recovery definition based on timing. Distinction between transient AKI/rapid reversal and persistent AKI appears to be clinically relevant as surrogate outcome variable for diagnostic testing in critically ill. Our results suggest also that partial recovery, rather than full renal recovery, may be also a clinically relevant signal which may deserve further research in this field.

## Additional file

**Additional file 1.** Quality of the dataset. **Table S1**: Discharge alive and 28-day mortality cause-specific model according to renal recovery defined as a decrease of at least one KDIGO class compared to the previous day. **Table S2**: Discharge alive and 28-day mortality cause specific model according to renal recovery defined as full recovery of AKI **Table S3**: Cox model of 28 day-mortality according to transient AKI defined as renal recovery occurring within the first 3 days as compared to persistent AKI.
