# Supplementary material for: ICU survival and need of renal replacement therapy with respect to AKI duration in critically ill patients
Source: Ann Intensive Care. 2018 Dec 17;8:127. doi: 10.1186/s13613-018-0467-6 (PMC6297118; doi:10.1186/s13613-018-0467-6)
Supplement: Supplementary file 1 — Additional file 1. Quality of the dataset. Table S1: Discharge alive and 28-day mortality cause-specific model according to renal recovery defined as a decrease of at least one KDIGO class compared to the previous day. Table S2: Discharge alive and 28-day mortality cause specific model according to renal recovery defined as full recovery of AKI Table S3: Cox model of 28 day-mortality according to transient AKI defined as renal recovery occurring within the first 3 days as compared to persistent AKI. [file 13613_2018_467_MOESM1_ESM.docx]

**Supplemental digital content**

**ICU SURVIVAL and NEED of RENAL REPLACEMENT THERAPY with respect to AKI DURATION in CRITICALLY ILL PATIENTS**

A.S. Truche ; S. Perinel Ragey, M.D.; B. Souweine, M.D., Ph.D. ; S. Bailly Pharm.D., Ph.D. ; L. Zafrani, M.D., Ph.D.; L. Bouadma, M.D, Ph.D.; C. Clec’h, M.D.; M. Garrouste-Orgeas, M.D., Ph.D.; G. Lacave, M.D.; C. Schwebel, M.D., Ph.D; F. Guebre-Egziabher, M.D., Ph.D.; C. Adrie, M.D.; AS. Dumenil, M.D. ; Ph. Zaoui, M.D., Ph.D.; L. Argaud, M.D., Ph.D.; S. Jamali, M.D.,Ph.D.; D. Goldran Toledano, M.D.; G. Marcotte, M.D.; JF. Timsit, M.D., Ph.D; M. Darmon, M.D., Ph.D.

*Quality of the database*

For most of the study variables, the data-capture software immediately ran an automatic check for internal consistency, generating queries that were sent to the ICUs for resolution before incorporation of the new data into the database. In each participating ICU, data quality was checked by having a senior physician from another participating ICU reviewing a 2% random sample of the study data on alternate years. A 1-day-long data-capture training course held once a year was open to all OUTCOMEREA^TM^ investigators and study monitors. All qualitative variables used in the analyses had κ coefficients >0.8 and all variables had inter-rater coefficients in the 0.67-1 range, indicating good to excellent reproducibility.

**Table S1: Discharge alive and 28-day mortality cause-specific model according to renal recovery** **defined as a decrease of at least one KDIGO class compared to the previous day, subgroups analyses.**

| **Parameter** | **Discharge alive** | | **28-Day mortality** | |
| --- | --- | --- | --- | --- |
|  | **CSHR (95% CI)** | **P value** | **CSHR (95% CI)** | **P value** |
| **Hypertension (n=1123)** | 1.75 (1.51-2.04) | <.01 | 0.43 (0.29-0.62) | <.01 |
| **Diabetes (n=1098)** | 1.67 (1.44-1.94) | <.01 | 0.63 (0.44-0.89) | <.01 |
| **Septic shock (n=1849)** | 2.71 (2.32-3.16) | <.01 | 0.51 (0.41-0.64) | <.01 |

Abbreviations: CSHR, Cause specific hazard ratio; CI, Confidence interval

**Table S2: Discharge alive and 28-day mortality cause specific model according to renal recovery defined as disappearance of KDIGO AKI diagnostic criteria:**

| **Parameter** | **Discharge alive** | | **28-Day mortality** | |
| --- | --- | --- | --- | --- |
|  | **CSHR (95% CI)** | **P value** | **CSHR (95% CI)** | **P value** |
| Shock at admission | 0.93 (0.86-0.99) | 0.03 | 1.25 (1.11-1.41) | <.01 |
| KDIGO Day 1 |  | 0.57 |  | 0.39 |
| 1 | ref |  | ref |  |
| 2-3 | 1.02 (0.95-1.09) |  | 1.05 (0.94-1.18) |  |
| Nephrotoxic in the 5 previous days *ͣ* | 0.77 (0.71-0.83) | <.01 | 0.93 (0.82- 1.05) | 0.23 |
| Daily Cardiological SOFA *ͣ* |  | <.01 |  | <.01 |
| 0 | ref |  | ref |  |
| 1 | 0.86 (0.79-0.92) | <.01 | 2.16 (1.77-2.65) | <.01 |
| 2 | 0.75 (0.63-0.89) | <.01 | 1.99 (1.41-2.80) | <.01 |
| 3 | 0.51 (0.46-0.57) | <.01 | 1.86 (1.51-2.30) | <.01 |
| 4 | 0.44 (0.39-0.49) | <.01 | 3.22 (2.65-3.90) | <.01 |
| Daily Liver SOFA *ͣ* |  | <.01 |  | 0.04 |
| 0 | ref |  | ref |  |
| 1 | 0.94 (0.84-1.05) | 0.26 | 1.00 (0.83-1.21) | 0.99 |
| 2 | 0.81 (0.72-0.92) | <.01 | 1.11 (0.93-1.32) | 0.26 |
| 3 | 0.48 (0.36-0.65) | <.01 | 1.19 (0.90-1.55) | 0.22 |
| 4 | 0.77 (0.60-0.98) | 0.03 | 1.51 (1.16-1.97) | <.01 |
| Daily Respiratory SOFA *ͣ* |  | <.01 |  | <.01 |
| 0 | ref |  | ref |  |
| 1 | 0.71 (0.64-0.77) | <.01 | 0.78 (0.63-0.95) | 0.01 |
| 2 | 0.65 (0.60-0.70) | <.01 | 0.86 (0.73-1.02) | 0.09 |
| 3 | 0.36 (0.32-0.41) | <.01 | 1.22 (1.02-1.45) | 0.03 |
| 4 | 0.35 (0.27-0.44) | <.01 | 1.98 (1.60-2.44) | <.01 |
| Daily Coagulation SOFA *ͣ* |  | 0.05 |  | <.01 |
| 0 | ref |  | ref |  |
| 1 | 0.95 (0.87-1.04) | 0.24 | 0.97 (0.82-1.16) | 0.76 |
| 2 | 0.90 (0.81-0.99) | 0.04 | 1.06 (0.88-1.26) | 0.56 |
| 3 | 0.93 (0.81-1.08) | 0.34 | 1.47 (1.21-1.79) | <.01 |
| 4 | 0.73 (0.55-0.95) | 0.02 | 2.17 (1.68-2.80) | <.01 |
| Daily Neurological SOFA *ͣ* |  | <.01 |  | <.01 |
| 0 | ref |  | ref |  |
| 1 | 0.66 (0.59-0.74) | <.01 | 1.39 (1.14-1.70) | <.01 |
| 2 | 0.53 (0.47-0.61) | <.01 | 1.68 (1.38-2.03) | <.01 |
| 3 | 0.42 (0.36-0.49) | <.01 | 2.17 (1.79-2.62) | <.01 |
| 4 | 0.31 (0.25-0.38) | <.01 | 4.52 (3.84-5.32) | <.01 |
| Renal recovery *ͣ* | 1.66 (1.54-1.78) | <.01 | 0.55 (0.47-0.66) | <.01 |

*ͣ* all time dependent variables values are those of the previous day.

Abbreviations: CSHR, Cause specific hazard ratio; CI, Confidence interval; KDIGO, Kidney disease: improving global outcomes; SOFA, Sequential organ failure assessment

**Table S3: Cox model of 28 day-mortality according to transient AKI defined as renal recovery occurring within the first 3 days as compared to persistent AKI:** The fourth day is considered as T0 (n=3584)

| **Parameter** | **HR (95% CI)** |  | **P value** |
| --- | --- | --- | --- |
| Transient AKI *ͣ* | 0.80 (0.67 - 0.95) |  | 0.01 |
| Shock at admission | 0.96 (0.81 - 1.13) |  | 0.63 |
| Maximum Cardiological SOFA*ᵇ* |  |  |  |
| ≤2 | Ref |  |  |
| >2 | 1.07 (0.90 - 1.27) |  | 0.43 |
| Maximum respiratory SOFA*ᵇ* |  |  |  |
| ≤2 | Ref |  |  |
| >2 | 1.38 (1.18 - 1.60) |  | <.01 |
| Maximum Liver SOFA*ᵇ* |  |  |  |
| ≤2 | Ref |  |  |
| >2 | 1.78 (1.39 - 2.27) |  | <.01 |
| Maximum coagulation SOFA*ᵇ* |  |  |  |
| ≤2 | Ref |  |  |
| >2 | 1.39 (1.14 - 1.69) |  | <.01 |
| Maximum neurological SOFA*ᵇ* |  |  |  |
| ≤2 | Ref |  |  |
| >2 | 1.91 (1.64 - 2.22) |  | <.01 |
| Maximum KDIGO class*ᵇ* |  |  |  |
| 1 | Ref |  |  |
| 2-3 | 1.02 (0.86 - 1.21) |  | 0.85 |
| Nephrotoxic*ᵇ* | 0.85 (0.73 - 1.00) |  | 0.05 |

*ͣ* Defined as an AKI lasting less than 3 days.

*ᵇ* in the first three days of ICU stay for persistent AKI and before renal recovery for transient AKI.

Abbreviations: HR, Hazard ratio; CI, Confidence interval; AKI, Acute kidney injury; SOFA, Sequential organ failure assessment; KDIGO, Kidney disease: improving global outcomes
